# Alanine Aminotransferase and Body Composition in Obese Men and Women

**DOI:** 10.1155/2019/1695874

**Published:** 2019-08-26

**Authors:** Svein Ivar Bekkelund, Rolf Jorde

**Affiliations:** ^1^Department of Clinical Medicine, UiT-The Arctic University of Norway, Tromsø, Norway; ^2^Department of Neurology and Neurophysiology, University Hospital of North Norway, Tromsø, Norway; ^3^Division of Internal Medicine, University Hospital of North Norway, N-9037 Tromsø, Norway

## Abstract

There is a known relationship between serum alanine aminotransferase (ALT) and obesity in humans, but the mechanism(s) are not clarified. This study investigated the associations between serum ALT and body composition in an overweight and obese population. The results are based on data from a previous randomized controlled trial treating obesity with vitamin D_3_. A sample of 448 overweight and obese individuals underwent dual-energy X-ray absorptiometry (DEXA) and measured serum ALT along with supplementary blood samples at study baseline. Body fat mass and lean mass indexes were calculated by dividing total body fat/lean weight (kg) by body height squared (kg/m^2^). ALT correlated with body mass index (BMI) in men but not women (*r* = 0.33, *P* < 0.0001 vs. *r* = 0.06, *P* = 0.29). In men, serum ALT correlated positively with fat mass index (*r* = 0.23, *P* = 0.004) and lean mass index (*r* = 0.32, *P* < 0.0001). In women, ALT correlated with lean mass index (*r* = 0.13, *P* = 0.031) but not fat mass index (*r* = 0.003, *P* = 0.96). In a multivariate model adjusted for age and fat mass index, a 1-unit increase in lean mass index associated with a 0.37 U/L higher ALT in the male subgroup (95% CI 0.024 to 0.040, *P* < 0.0001). In conclusion, serum ALT was associated with body fat mass index in men and with lean mass index in men and women in an overweight and obese population. The findings also demonstrate a gender difference in the role of fat.

## 1. Introduction

Alanine transaminase (ALT) is associated with obesity [1, 2], cardiovascular disease (CVD), and CVD-related mortality [3–5]. Clinical and population studies have related ALT with insulin resistance, metabolic syndrome, and type 2 diabetes [6–8]. Obesity is reported to be a major risk factor to develop nonalcoholic fatty liver disease (NAFLD), a common liver disease defined as ≥5% fat liver [9], and an important mechanism behind the relationship between ALT and risk of CVD [10, 11]. Hepatic accumulation of lipids occurs initially while inflammation and oxidative stress in response to increased lipid activity are further reaction characteristic for the disease [12, 13].

A Korean population study showed a higher risk of elevated ALT by increasing degree of BMI [14]. Odds ratio for elevated ALT in obese subjects was 5.0 in men and 3.9 in women [14]. In studies from the United States confirming a positive relationship between ALT and BMI, the strongest association was found for waist-to-hip ratio [15] and trunk fat using dual-energy X-ray absorptiometry (DEXA) to measure body composition, indicating central adiposity to be an important obesity-related determinant of elevated ALT [16]. ALT was also associated with trunk lean mass in both sexes [16].

Little is known about the muscular role of ALT in general, and obesity in particular, but the parallel increase and the following recovery of ALT released to the circulation in response to muscle injury, seizure, and inflammation may reflect some underlying mechanisms [17, 18]. There is emerging evidence that loss of muscle mass (sarcopenia) plays a role in the complex pathophysiology of NAFLD [19]. One link between them is insulin resistance, which is associated with muscle loss [20]. Furthermore, in a retrospective cohort, age-related reduction in skeletal muscle mass and increase in fat mass over time were associated with incidents of NAFLD, although only in nonobese subjects [21]. The relative contribution of fat mass and lean mass to the relationship with ALT and how it integrates with obesity in presumptive healthy obese individuals is necessary supplementary knowledge to obtain. The study purpose was to investigate the relationship between ALT and body composition in an overweight and obese population.

## 2. Material and Methods

### 2.1. Study Participants

Using data from a randomized controlled trial (RCT) treating obesity with vitamin D_3_ [22], the present study was designed with the following criteria: males and females 21-70 years of age with BMI between 28.0 and 47.0 kg/m^2^ were accepted for inclusion. Diabetes, history of heart infarction, angina pectoris and stroke, weight loss > 10 kg last six months, use of antidepressants and drugs with weight-reducing properties, participation in weight loss programs, pregnant and lactating women, women with pregnancy plans next 12 months, women < 50 years without use of contraceptives, males with serum creatinine > 129 *μ*mol/L, and females with serum creatinine > 104 *μ*mol/L were the exclusion criteria. The oral glucose tolerance test was performed in all participants to detect unrecognized diabetes. All participants recruited from this general population were Caucasians. No systemic disorders including malignancies, alcoholism, hepatitis or other hepatic disorders, or use of hepatotoxic medication were identified in any subjects. The participants were either recruited via newspaper advertisement or from the medical outpatient clinic at the University Hospital of North Norway. Written consent was obtained from all, and the Norwegian Committee for Medical and Health Research Ethics (REC) approved the study. The study was conducted in accordance with the Helsinki Declaration.

### 2.2. Measurements

Only baseline data were used. Standardized measurements of height and weight were performed with light clothing without shoes, and body mass index (BMI) was calculated as weight (kg) divided by height squared (m^2^). Body composition measurements with DEXA (GE Lunar Prodigy, LUNAR Corporation, Madison, WI, USA) were performed, and body fat index was calculated by dividing total body fat weight (kg) by body height squared (m^2^). The same was done for lean mass. Physical activity, a potential confounder, was calculated by the short version (7 days) of the International Physical Activity Questionnaire (IPAC) [23]. Vigorous, moderate, and walking activities are transformed to units of metabolic equivalents (MET)-min/week, where METs are multiples of the resting metabolic rates.

Serum ALT was analyzed consecutively within 6 hours after the phlebotomies in an automated clinical chemistry analyzer (Modular P, Roche) by photometry, using an enzymatic method (CK-NAC, Roche Diagnostics, Mannheim, Germany). Reference limits for serum ALT were 10-70 U/L (men) and 10-45 U/L (women). The lower detection limit of ALT assay was 5.0 U/L. The analytical variation (Vka) of ALT is 4.9%. The standard cut-off limits for ALT and AST used in the hospital are developed by the Nordic Reference Interval Project (NORIP) [24]. AST/ALT ≥ 2 and ≥3 were calculated to detect participants at risk of alcoholic liver disease [25]. Gamma-glutamyl transpeptidase (GGT) was measured with references 10-80 U/L (men 18-39 years), 15-115 U/L (men ≥ 40 years), 10-45 U/L (women 18-39 years), and 10-75 U/L (women ≥ 40 years) with 3.2% Vka. Nonfasting S-glucose and glycosylated hemoglobin (HbA_1C_) in EDTA whole blood based on an immune turbidometric assay (UNIMATES, F. Hoffmann-La Roche AG) were obtained. The HbA_1C_% was calculated from the HbA_1C_/Hb ratio. Serum total cholesterol was analyzed by an enzymatic colorimetric method using a commercially available kit (CHOD-PAP, Boehringer-Mannheim, Mannheim, Germany). All analyses were done at the Department of Clinical Biochemistry, University Hospital of North Norway.

### 2.3. Statistical Analysis

The statistical analysis was performed by SPSS software version 25 (SPSS INC., Chicago, Illinois, USA). Distributions of the data were reviewed by visual inspection of histograms and by calculation of kurtosis and skewness. The histograms showed right-sided skewness in all endpoint variables. Serum ALT (skewness 2.7, kurtosis 4.0) and serum AST (skewness 4.2, kurtosis 37.3) confirmed a non-Gaussian distribution of the data. Log-transformed data were normal-distributed and therefore used in the analyses. The analyses were performed sex-stratified since levels of ALT and components of body composition are different in men and women. Descriptive data are presented as mean ± standard deviations (SD) or numbers and frequencies. Two-sided Student's *t*-test was used to calculate differences between means and ANOVA used to compare body composition with quartiles of ALT (analyses of trends). The *χ*^2^ test was used to compare frequencies of data within groups (dichotomous data). By multiple regression analysis, possible confounders were tested and adjusted for with ALT as dependent variables, and variables that significantly correlated with ALT were included in the regression model as independent variables. Regression coefficients (ß) with 95% confidence interval (CI) were calculated. The level of significance was set at ≥5%.

## 3. Results

**Figure 1:**
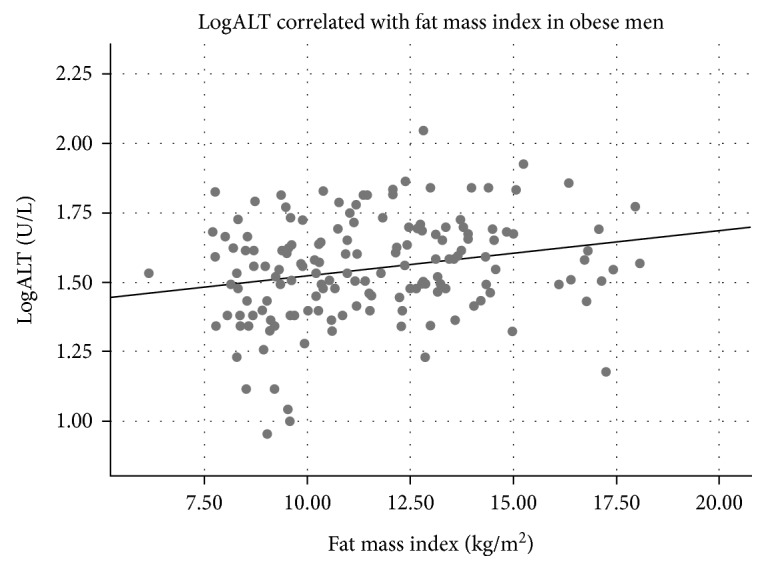
Correlation between alanine aminotransferase (ALT) and fat mass in 157 obese men (*r* = 0.23, *P* = 0.004).

**Figure 2:**
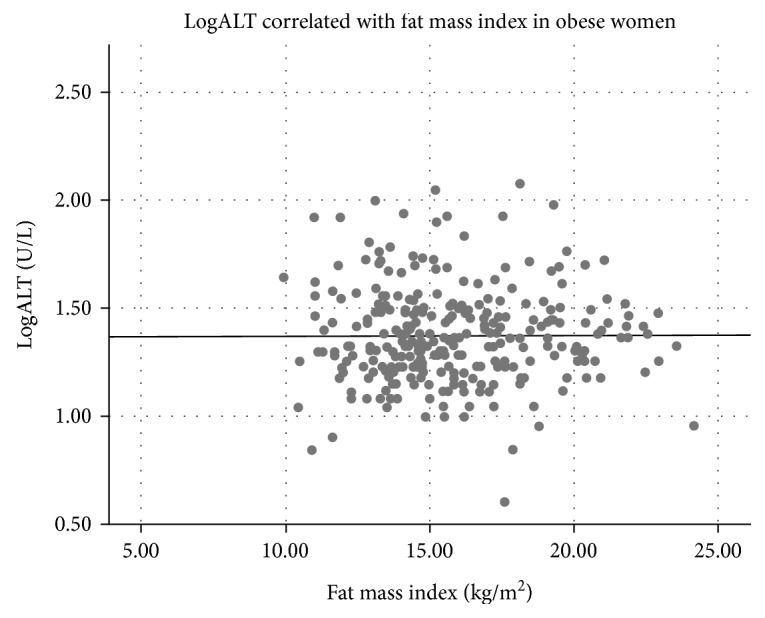
Correlation between alanine aminotransferase (ALT) and fat mass in 291 obese women (*r* = 0.003, *P* = 0.96).

**Figure 3:**
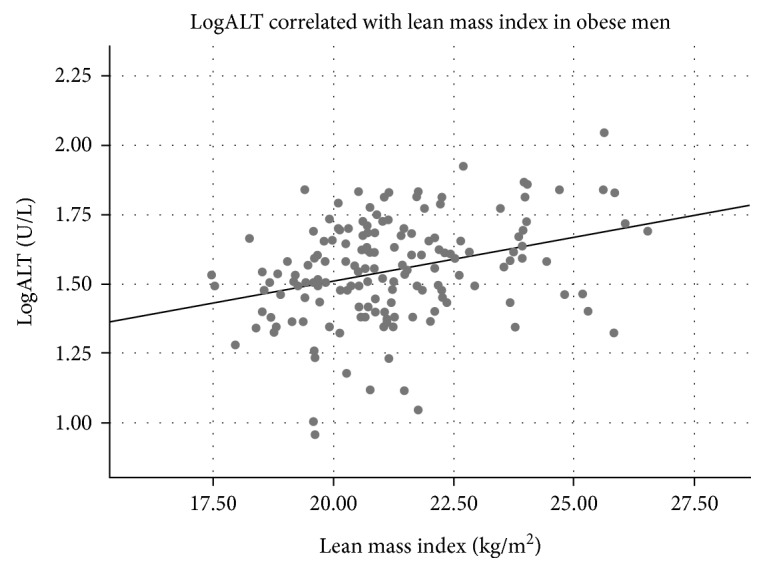
Correlation between alanine aminotransferase (ALT) and lean mass in 157 obese men (*r* = 0.32, *P* < 0.0001).

**Figure 4:**
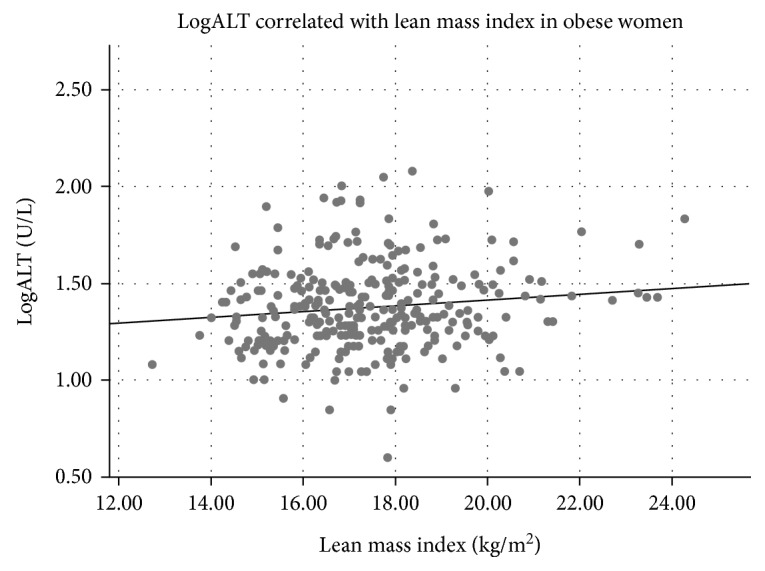
Correlation between alanine aminotransferase (ALT) and lean mass in 291 obese women (*r* = 0.13, *P* = 0.031).

Clinical variables of the study population are listed in Table 1. A majority of the participants were obese (BMI ≥ 30 kg/m^2^), while about 10% of both sexes were overweighed (25 ≥ BMI < 30 kg/m^2^) (Table 1). ALT was associated with BMI in men but not in women (Table 2), and BMI was significantly associated with ALT quartiles in men (Table 3). In contrast to women, ALT correlated positively with fat mass index in men and with lean mass indexes in both sexes (Table 2, Figures 1–4). ALT was significantly correlated with serum glucose, HbA_1C_, and cholesterol in women but not in men (Table 2). The highest ALT value was 120 U/L (woman) and the highest AST was 137 U/L (woman). Nine participants (3 men and 6 women) had AST/ALT index ≥ 2 and 2 women had AST/ALT index ≥ 3.

Fat mass and lean mass indexes increased significantly with increasing quartiles of serum ALT in men in a trend analysis using ANOVA (Table 4). In contrast, lean mass but not fat mass indexes increased significantly from quartiles 1 to 4 of serum ALT in women (Table 3). Lean mass index was inversely and independently associated with serum ALT when adjusted for covariates in men (Table 4). Furthermore, a 1-unit increase in lean mass was associated with 0.37 U/L higher serum ALT when adjusted for age and fat mass index (Table 4). This association was independent and significant also when replacing body composition variables with BMI (data not shown).

## 4. Discussion

ALT was log-linearly and positively associated with fat mass index and lean mass index in men and with lean mass index in women in an obese cohort. After adjusting for obesity-related variables, body lean mass index remained independently associated with ALT in the male subgroup. ALT may hypothetically play a favourable role in the adipose process, but the fat mass component of body composition may act differently in women.

Although ALT is mainly located in the liver, alanine synthesis also occurs in muscle tissue [26, 27]. A parallel increase and recovery after muscular strain in both ALT and in the energy reactive muscle enzyme creatine kinase (CK) are reported [18]. In patients with rhabdomyolyses (CK ≥ 1000 U/L), 75% had abnormal ALT, which confirms its muscular involvement [28]. In contrast to ALT, CK was not associated with muscle mass in obese subjects measured at rest indicating different muscular relationships between them [29]. Whether ALT plays a positive or negative role, or both, in the CVD processes is discussed in the literature. Sarcopenia (age-related loss of muscle mass and strength) is an area of research where the ALT-muscular relationship has been questioned. Both adiposity and sarcopenia share in common an increased risk of NAFLD, and both have been associated with ALT and insulin resistance [14, 21, 30]. Furthermore, increased ALT may predict reduced insulin sensitivity and diabetes [6, 7]. The mechanisms are complex and incompletely understood, but the relationship between ALT and muscle mass as well as insulin resistance may be mediated by inflammation. ALT was associated with low-graded inflammation (CRP) in 1483 middle-aged Japanese men, and proinflammatory cytokines may exert a negative (catabolic) muscular effect [31, 32]. Additionally, insulin resistance in skeletal muscle may be produced by inflammatory activity such as tumor necrosis factor alpha and complement 3 [33, 34]. It is previously known that obesity-related inflammation stimulate progression of NAFLD and development of insulin resistance [12, 13]. Lack of relationship between ALT and CRP in the present study does not exclude the influence of inflammatory metabolites, however.

The Korean sarcopenia obesity study reported recently a 5.2-time increased risk of NAFLD in the sarcopene obese group compared to nonobese [30]. An inverse correlation between ALT and skeletal muscle mass index as well as total body fat and additionally negative correlations with CRP, components of metabolic syndrome, and LDL-cholesterol were reported [30]. Moreover, ALT was inversely associated with sarcopenia, CVD, and overall mortality in 765 elderly subjects analyzed in prospective population-based data [35]. ALT correlated with appendicular lean mass, trunk lean mass, and total lean mass but not fat mass in a group of 174 healthy young athletic women [36]. A US population-based study measuring body mass with DEXA showed an association between lower ALT and mortality risk [37]. Lower values of appendicular lean mass were found in the three lowest ALT centiles when adjusted for total body fat mass, which could possibly explain the lower mortality rate in those with low ALT values [37]. In line with this, ALT was inversely and linearly associated with CVD risk in a 10.5-year follow-up study that included 6899 participants and 729 CVD events [38]. We did not measure partial body composition such as appendicular lean mass, neither was muscular power examined. Highly significant associations between ALT and total body lean mass in the present study in otherwise healthy obese individuals make that explanation less likely. ALT, by its muscular connection, may hypothetically play a beneficial role in the adipose process. The interrelationships between ALT, NAFLD, lean mass, and inflammation should be targeted in future studies.

ALT is connected with adiposity and CVD risk factors [39]. A positive association between ALT and trunk fat independent of trunk and extremity lean mass was found in one study [16], while central adiposity along with generalized adiposity is reported by others [5]. Furthermore, ALT was not elevated in otherwise healthy obese people in a clinical study with limited number of participants contrasting our results [40]. This corresponds approximately with the relatively small subgroup with elevated ALT frequency (about 8%) found in the present study. Overall, relationships between ALT and CVD risk are complex [41, 42]. Additionally, how lean mass and fat mass act in the atherosclerotic process is not clear. Lean mass was independently associated with carotid media thickness in 421 obese subjects [43] and was associated with carotid lumen diameter in another study [44]. On the other hand, lean mass predicted a better cardiac function in a 10-year follow-up study of obese subjects indicating a protective CVD effect [45].

As found in the present study, ALT is reported to be higher in males than females [46, 47]. Whether this is due to different fat vs. lean mass contribution, the effect of sex hormones or other mechanisms is not known. The positive relationship between ALT and the muscular component relative to fat in the female group of the present obese body composition sample illustrates the complexity of body composition and its connection with ALT. Whether ALT may play a beneficial role in the adipose female process is an open question. Thus, ALT predicted coronary heart disease in men but not in women in a European-American population-based study [48]. Further, a link between ALT and muscular glucose uptake was found in women only, which may hypothetically explain why ALT appears to play a different role as a CVD risk marker in men and women [49]. In parallel, ALT correlated with metabolic variables (glucose, HbA_1C_, and cholesterol) in women but not in men in the present study. In a large Italian study, ALT associated positively with BMI, glucose, cholesterol, and triglycerides and increased with younger age groups (until third decade in males and fifth decade in females) but decreased in older age groups [50]. Consequently, gender should be taken into account when planning clinical ALT studies.

### 4.1. Strengths and Shortcomings

Although the risk of statistical type 2 error is higher in secondary research studies due to uncertain sample size and invalid selection criteria, the larger female subgroup provides evidence to support the findings of gender differences in this study. BMI do not distinguish between fat and muscle content, nor does it reflect body fat distribution. These characteristics may impair the validity of BMI as an obesity marker and argue for the use of DEXA in such studies [51, 52]. Similarly, people with sarcopenic phenotype, i.e., those with increased adipose tissue and reduced muscle mass, may be overlooked by BMI [52]. Physical activity score did not alter the results here but is a potential confounder to consider in such studies [18, 53]. A drawback to the study is lack of information about alcohol consumption since ALT and AST are not sensitive alcohol markers [54].

## 5. Conclusion

ALT was positively associated with body lean mass in men and women but associated with fat mass only in men in this obese population. These findings suggest that the ALT-obesity relationship may partly be explained by different gender biology. Whether lean mass is more important than fat mass to explain how ALT relates to obesity needs to be confirmed and further investigated.

## Figures and Tables

**Table 1:** Clinical characteristics of the subjects. Numbers (%) or mean (SD) is presented.

Variables	Total group (*n* = 448)	Men (*n* = 157)	Women (*n* = 291)	*P*
Age (years)	47.5 (11.4)	47.8 (10.8)	47.4 (11.8)	0.15
Use of antihypertensive drugs	93 (20.8)	33 (21.0)	60 (20.6)	1.0
Statin use	43 (9.6)	12 (7.6)	31 (10.7)	1.0
NSAID	74 (16.5)	27 (17.2)	47 (16.2)	1.0
H2-blocking drugs	14 (3.1)	3 (1.9)	11 (3.8)	1.0
Antidepressants	25 (5.6)	10 (6.4)	15 (5.2)	0.52
Height (cm)	169.3 (8.9)	178.3 (6.1)	164.5 (6.0)	0.76
Weight (kg)	99.3 (14.2)	109.2 (11.2)	94.0 (11.7)	0.020
BMI (kg/m^2^)	34.6 (3.9)	34.3 (3.6)	34.7 (4.1)	0.15
Obesity (BMI ≥ 30 kg/m^2^)	406 (90.6)	141 (89.8)	265 (91.1)	0.73
Overweight (25 ≥ BMI < 30 kg/m^2^)	42 (9.4)	16 (10.2)	26 (8.9)	1.0
Fat mass (kg)	40.6 (8.3)	36.7 (8.4)	42.6 (7.6)	0.018
Lean mass (kg)	54.2 (11.7)	67.5 (7.4)	47.1 (5.9)	0.027
Fat mass index (kg/m^2^)	14.3 (3.4)	11.5 (2.6)	15.8 (2.8)	<0.0001
Lean mass index (kg/m^2^)	18.7 (2.6)	21.2 (1.9)	17.4 (1.9)	<0.0001
ALT (U/L)^∗^	31.2 (17.5)	38.5 (15.9)	27.3 (17.1)	<0.0001
High ALT^∗∗^	38 (8.5)	4 (2.5)	34 (11.7)	0.001
AST (U/L)^∗^	25.7 (10.0)	29.0 (8.7)	24.0 (10.3)	0.72
High AST^∗∗^	37 (8.3)	12 (7.6)	25 (8.6)	0.86
GGT (U/L)	32.0 (38.8)	37.2 (39.2)	27.4 (37.9)	<0.0001
Creatine kinase (U/L)^∗^	121.6 (120.3)	178.5 (180.7)	91.0 (46.3)	<0.0001
S-glucose (mmol/L)	5.35 (0.64)	5.47 (0.59)	5.27 (0.64)	0.92
S-HbA1c (%)	5.66 (0.38)	5.67 (0.42)	5.65 (0.36)	0.14
Hs-CRP (mg/dL)^∗^	4.08 (4.87)	3.30 (4.71)	4.45 (4.82)	0.08
S-total cholesterol (mmol/L)	5.37 (1.00)	5.40 (0.89)	5.36 (1.06)	0.022
Physical activity score (MET-min/week)^∗^	3207.0 (3836.0)	3046.7 (4273.2)	3297.8 (3587.4)	<0.0001

BMI: body mass index; ALT: alanine transaminase; AST: aspartate aminotransferase; GGT: gamma-glutamyl transpeptidase; Hs-CRP: high sensitive C-reactive protein; MET: metabolic equivalent; NSAID: nonsteroid antiflogistic drugs. ^∗^Analyzed log-transformed. ^∗∗^Above reference limit.

**Table 2:** Correlations between ALT^∗^, body composition, and potential confounders.

	ALT (U/L) Men (*n* = 157)		ALT (U/L) Women (*n* = 291)	
	*r*	*P*	*r*	*P*
Age (years)	-0.19	0.017	0.23	<0.0001
BMI (kg/m^2^)	0.33	<0.0001	0.006	0.29
Fat mass index (kg/m^2^)	0.23	0.004	0.003	0.96
Lean mass index (kg/m^2^)	0.32	<0.0001	0.13	0.031
Creatine kinase (U/L)^∗^	0.15	0.07	0.06	0.35
S-glucose (mmol/L)	0.09	0.27	0.23	<0.0001
S-HbA_1C_ (%)	0.11	0.18	0.19	0.001
Hs-CRP (mg/dL)^∗^	0.06	0.44	-0.01	0.84
S-total-cholesterol (mmol/L)	0.02	0.86	0.16	0.005
Physical activity score (MET-min/week)^∗^	-0.09	0.31	-0.02	0.80

BMI: body mass index; ALT: alanine transaminase; Hs-CRP: high sensitive C-reactive protein; MET: metabolic equivalent. ^∗^Analyzed log-transformed.

**Table 3:** Fat mass index, lean mass index, and confounders in quartiles of serum ALT.

ALT^∗^ quartiles					
	Q1	Q2	Q3	Q4	*P* for trend
Q-intervals (U/L) (men)	≤1.42	1.43-1.54	1.55-1.66	≥1.67	
*N* = 157	37	40	38	42	
Age (years)	51.1 (11.9)	48.8 (11.8)	45.6 (9.6)	45.9 (10.0)	0.09
BMI (kg/m^2^)	32.7 (3.3)	33.8 (3.5)	34.7 (3.0)	36.0 (3.5)	<0.0001
Fat mass index (kg/m^2^)	10.4 (2.2)	11.7 (2.6)	11.6 (2.6)	12.3 (2.4)	0.008
Lean mass index (kg/m^2^)	20.7 (1.7)	20.7 (1.8)	21.5 (1.5)	22.2 (2.0)	<0.0001
Q-intervals (U/L) (women)	≤1.22	1.23-1.33	1.34-1.48	≥1.49	
*N* = 291	69	72	71	79	
Age (years)	42.4 (12.1)	45.7 (12.0)	50.6 (12.4)	51.0 (9.8)	<0.0001
BMI (kg/m^2^)	33.9 (3.8)	34.6 (3.9)	35.7 (4.3)	34.7 (4.2)	0.62
Fat mass index (kg/m^2^)	15.5 (2.7)	15.5 (2.9)	16.6 (2.9)	15.5 (2.7)	0.10
Lean mass index (kg/m^2^)	16.9 (1.7)	17.4 (1.6)	17.5 (2.1)	17.7 (1.9)	0.078
S-glucose (mmol/L)	5.06 (0.60)	5.19 (0.55)	5.45 (0.71)	5.37 (0.60)	0.001
S-HbA1C (%)	5.54 (0.34)	5.61 (0.36)	5.76 (0.36)	5.67 (0.34)	0.003
S-total-cholesterol (mmol/L)	5.02 (0.94)	5.18 (1.11)	5.63 (1.04)	5.54 (1.03)	0.001

ALT: alanine transaminase; Q1: first quartile; Q2: second quartile; Q3: third quartile; Q4: fourth quartile. ^∗^Analyzed log-transformed.

**Table 4:** Associations between ALT^∗^ (dependent variable) and independent variables in overweight and obese men and women.

	ALT (U/L) as dependent variable		
	ß^∗∗^	*95% CI*	*P*
Men (*n* = 157)			
Age (years)	0.12	0.001 to 0.004	0.008
Fat mass index (kg/m^2^)	0.06	-0.10 to 0.002	0.18
Lean mass index (kg/m^2^)	0.37	0.024 to 0.040	<0.0001
Adjusted *R*^2^	0.16		
Women (*n* = 291)			
Age (years)	0.15	0.000 to 0.005	0.034
Lean mass index (kg/m^2^)	0.11	0.000 to 0.027	0.051
S-glucose (mmol/L)	0.17	0.014 to 0.113	0.01
S-HbA1c (%)	0.04	-0.063 to 0.120	0.54
S-total-cholesterol (mmol/L)	0.06	-0.130 to 0.40	0.31
Adjusted *R*^2^	0.09		

ALT: alanine transaminase. ^∗^Analyzed log-transformed. ^∗∗^Values are regression coefficients (95% CI) expressed in ALT U/L for a 1-unit change in independent variables.

## Data Availability

The data used to support the findings of this study are available from the corresponding author upon request.
